# Mapping quantitative trait loci (QTL) in sheep. IV. Analysis of lactation persistency and extended lactation traits in sheep

**DOI:** 10.1186/1297-9686-43-22

**Published:** 2011-06-21

**Authors:** Elisabeth Jonas, Peter C Thomson, Evelyn JS Hall, David McGill, Mary K Lam, Herman W Raadsma

**Affiliations:** 1 ReproGen-Animal Bioscience Group, Faculty of Veterinary Science, University of Sydney, 425 Werombi Road, Camden NSW 2570, Australia

## Abstract

**Background:**

In sheep dairy production, total lactation performance, and length of lactation of lactation are of economic significance. A more persistent lactation has been associated with improved udder health. An extended lactation is defined by a longer period of milkability. This study is the first investigation to examine the presence of quantitative trait loci (QTL) for extended lactation and lactation persistency in sheep.

**Methods:**

An (Awassi × Merino) × Merino single-sire backcross family with 172 ewes was used to map QTL for lactation persistency and extended lactation traits on a framework map of 189 loci across all autosomes. The Wood model was fitted to data from multiple lactations to estimate parameters of ovine lactation curves, and these estimates were used to derive measures of lactation persistency and extended lactation traits of milk, protein, fat, lactose, useful yield, and somatic cell score. These derived traits were subjected to QTL analyses using maximum likelihood estimation and regression analysis.

**Results:**

Overall, one highly significant (LOD > 3.0), four significant (2.0 < LOD < 3.0) and five suggestive (1.7 < LOD < 2.0) QTL were detected across all traits in common by both mapping methods. One additional suggestive QTL was identified using maximum likelihood estimation, and four suggestive (0.01 < P < 0.05) and two significant (P < 0.01) QTL using the regression approach only. All detected QTL had effect sizes in the range of 0.48 to 0.64 SD, corresponding to QTL heritabilities of 3.1 to 8.9%. The comparison of the detected QTL with results in cattle showed conserved linkage regions. Most of the QTL identified for lactation persistency and extended lactation did not coincide. This suggests that persistency and extended lactation for the same as well as different milk yield and component traits are not controlled by the same genes.

**Conclusion:**

This study identified ten novel QTL for lactation persistency and extended lactation in sheep, but results suggest that lactation persistency and extended lactation do not have a major gene in common. These results provide a basis for further validation in extended families and other breeds as well as targeting regions for genome-wide association mapping using high-density SNP arrays.

## Background

Lactation performance plays an important role for the productivity and therefore economic value of dairy cattle and dairy sheep. Persistency of lactation is a trait of considerable importance as it reflects the ability of an animal to maintain milk production at a high level after the peak yield [1]. It has been found that cows with greater lactation persistency tend to incur lower feed, health, and reproductive costs [2], and cows affected with mastitis tend to have less persistent lactations [3]. The animal health benefits of increased lactation persistency are speculative at present; but it has been hypothesised that increased lactation persistency is associated with lower incidences of peri-parturient metabolic diseases [4]. With the recent interest in year-round calving as opposed to the conventional 305-day cycle in dairy cattle, identifying the genetic basis for lactation persistency and extended lactation is of interest.

Lactation curve models have had a long history in dairy science, dating back at least to Brody [5]. Since then, many models have been proposed, including the commonly used model by Wood [6], which differ both in the mathematical form of the function and the number of model parameters that need to be estimated. More recently, random regression approaches have been proposed, which allow a model to be fitted to the yield data in which the data itself specifies the shape of the fitted model, rather than fitting to a pre-specified mathematical function [7,8]. However, random regression models require a large number of lactations with many yields recorded per lactation, which may be a problem in voluntary recording systems. Furthermore, extraction of summary information from fitted random regression models is difficult.

As well as being able to predict lactation yield at any stage of lactation, lactation curve models can be used to summarise key features of the lactation curve, such as time of peak yield and the peak flow, and characteristics related to extended lactation. One of the difficulties with some of these lactation length measures is the variety of definitions provided in the literature (e.g. Sölkner and Fuchs [2]), although there are some common threads. Lactation persistency is usually defined as the (rate of) decline of milk yield after the peak [6,9]. Lactation persistency has also been defined as the difference in yield between peak to a defined day later in lactation [10]. However, Grossman et al. [11] developed a new model for lactation curves which allows the definition of persistency as the number of days at peak lactation. However, the models developed by Grossman et al. [11] implied a lactation curve with a plateau, which is not the case for the typical lactation curve in sheep which has a relatively early but sharp peak. These derived measures of lactation persistency can then be used as traits in QTL mapping studies [12,13].

Compared with lactation persistency, extended lactation has received relatively less attention in the literature. Extended lactation deals with the ability to maintain lactation at productive levels beyond the usual "drying off' period or a fixed reference day, i.e. beyond 305 days in cattle, or 100 days in dairy sheep. Both parametric and semi-parametric models can be used to quantify extended lactation. They all consider the level of milk production beyond the reference day. Some studies fit models to empirical data from extended lactation records [14], whilst others use fitted models to predict the potential for extended lactation in the absence of actual data records beyond the reference date. Vargas et al. [15] analysed the accuracy of different models to predict daily milk yield in standard and extended lactations in cattle. Dematawewa et al. [16] compared empirical (including the Wood model) and mechanistic lactation models for their suitability to predict standard (305-day) and extended (999-day) lactations in cattle (with 0.1% of the cows having actual data on the extended lactation). They recommended the use of the model of Rook et al. [17] or the Wood model as being appropriate to describe extended lactations including milk, fat and protein yields [16]. Previously, we showed that the Wood model was appropriate to describe lactation curves in sheep [18], so here we extend the use of this model to predict lactation persistency and extended lactation in sheep.

To our knowledge, there are no reported heritability estimates for lactation persistency in sheep, nor for extended lactation in sheep or cattle. Reported heritability estimates for milk persistency in cattle were between 0.02 and 0.27 [19]. Heritability estimates for milk composition and somatic cell count persistency were between 0.03 and 0.2 [4,20]. Thus, under conventional mass selection, the rate of gain for persistency of milk yield and milk composition is expected to be low.

Growing intensification of sheep and goat production systems during the recent decades have led to increased research interests into the yield and composition of milk from small ruminants [21-23]. Milk yield and milk components (%) are negatively correlated in sheep and these correlations change during the lactation [24,25]. It has been shown that the quality of cheese processing also declines during the course of lactation in sheep with poor lactation persistency [26]. Changes in milk composition of dairy sheep throughout the lactation typically result in variable cheese yields and quality [27]. Selecting animals with improved lactation persistency should therefore also aim at more persistent milk composition and yield during the course of the lactation. This would benefit the use of milk for processing into cheese and other products.

Until now, only a few linkage studies have been published for traits related to persistency of milk yield and milk composition in cattle but none have been reported in sheep. QTL were reported for milk, fat, protein or energy persistency [10,12,28] and shape of the lactation curve [29] in cattle. Comparison of results across studies is problematic as there is no universally accepted method to calculate persistency, and results are therefore method-dependent. The objective of this study was to report on an appropriate model to predict lactation persistency and extended lactation for milk yield and milk composition traits in sheep, and to provide additional information on QTL for these traits. The application of QTL information for these traits is of potential interest in marker-assisted selection since these traits have sex-limited expression, and are potentially difficult to measure, thus limiting the rate of progress under conventional mass selection.

## Methods

### Resource population and phenotypes

A resource population from crosses between the improved dairy type of Awassi (A) and the apparel wool Merino (M) sheep was established as described in detail by Raadsma et al. [30]. Genotypic information from the backcross progeny of the first sire was used for linkage mapping.

Lactation and milk composition records were collected for 172 backcross ewes between 1999 and 2007 [18]. Milk yield (MY) was recorded every second day, and protein, fat, lactose percentages and somatic cell count (SCC) were analysed from milk sampled once weekly. Somatic cell score (SCS) was calculated as the logarithm to base 10 of the raw SCC. The yields of protein (PY), fat (FY), lactose (LY) and somatic cell score (SCY) were calculated by multiplication of the contents (expressed as a percentage) with MY. Useful yield (UY) was calculated as FY + 1.85 × PY [31]. Lactation curves for MY, PY, FY, LY, UY and SCY were estimated by fitting Wood models [32] to the yield data. Due to its simplicity and reliable fit to the data, the Wood model was chosen as the preferred method to summarise lactation curve characteristics. The basic form of the Wood model is defined as follows:

where *W*(*t*) is the expected yield at time *t*, and *k *= ln(*a*), *b *and *c *are parameters controlling the shape of the curve. Specifically, *a *is related to the total area under of the curve, *b *is related to the sharpness of the early rise, and *c *describes the decline rate in milk production. Multiple lactation curves were fitted simultaneously using the nlme function in R, treating each ewe by lactation as a random effect. Further description of the Wood model parameters and procedure for model fitting is in Raadsma et al. [18].

From this model, the cumulative yield up to day *T *(e.g. 100 days) (CumY(*T*)) can be calculated numerically as , where γ(·) is the lower incomplete gamma function, . Furthermore, the day of the maximum yield (*t*_max_) and the maximum yield (maxY) were calculated as *t*_max _= *b*/*c *and maxY = *a*(*b*/*c*)*^a^e*^-*b *^respectively.

Milk persistency was defined as the expected milk yield on day *T*, relative to that on the peak day, namely PersY(*T*) = *W*(*T*)/maxY. A more persistent lactation will have a flatter curve, with the persistency proportion approaching one. In the same way, derived persistency variables were also calculated for PY, FY, LY, UY and SCY; furthermore, lactation persistency is defined as a general term for milk as well as persistency of milk composition.

A previous study showed that 100 day lactation performance, measured as cumulative milk yield until day 100, is highly correlated with cumulative milk yield to day 300, whereas lactation performance taken as either cumulative milk yield to day 50 or 80 was unreliable in predicting cumulative lactation performance [18]. Furthermore, the number of observations in this data set was still very high (*n *= 565) at day 100 of lactation, which reinforced the choice of evaluating persistency at *T *= 100 days [see Additional file 1].

Extended lactation deals with the ability of the ewe to sustain milk production beyond a certain time. In the absence of a standard lactation length, as in cattle and as outlined in paper II of this series [18], we have adopted day 100 as the standard reference for lactation length in sheep (in cattle this would nominally be day 305), even though this is shorter than most of the average ovine lactation lengths in the literature [33,34]. The definition of extended lactation adopted here is the ratio of expected production from day 100 to 300, relative to the cumulative production (of MY, PY, FY, LY, UY and SCY) up to day 100, i.e. [CumY(300)-CumY(100)]/CumY(100). The greater this ratio is, the more extended the lactation is. Note that the cut off at day 300 is arbitrarily made in order not to have an "infinite" lactation length.

### QTL mapping procedure

A genome-scan using 189 polymorphic microsatellite markers covering all 26 autosomes was conducted on 172 backcross ewes. The procedures of DNA extraction, genotyping, allele calling, and the linkage map have been described previously [30].

Based on a Type I error of 0.05, the design had a predicted power of 0.72 to detect QTL with 0.4 SD effect [35]. To achieve normality, all traits were log-transformed prior to using two methods for QTL analyses. Solutions were first obtained using a maximum likelihood procedure, named QTL-MLE in R [30,36], which is suitable for the analysis of a backcross population. For QTL-MLE, a LOD of 1.75-2.0 was deemed suggestive, LOD 2.0-3.0 significant, and LOD > 3.0 highly significant. The 1-LOD drop-off method was used to calculate the respective confidence intervals for this method. QTL effects were derived from the model and normalised against a model with no QTL to express the size of QTL effects in units of phenotypic standard deviations.

The second method for QTL detection was based on the regression analysis for a half-sib design using QTL Express [37]. The half-sib model was used since no maternal genotypes were available. For this method, QTL with chromosome-wide significance thresholds (*P *< 0.05) were described as suggestive, chromosome-wide levels *P *< 0.01 as significant and experiment-wide levels (*P *< 0.05) as highly significant QTL. A two-QTL model was also fitted to all data using the same program [37] and this was conducted over all chromosomes. Chromosome-wide significance thresholds were assessed using permutation tests, and bootstrap procedures were used to obtain confidence intervals, both implemented in QTL Express using 1,000 re-samplings. The QTL heritability was calculated as the proportion of the phenotypic variance accounted for by the QTL, and this is calculated from the residual mean squares from the regression analysis of fitting a QTL or no QTL as [1-(residual mean square of full model/residual mean square of reduced model)].

### Meta-assembly

To facilitate a comparative genome analysis between sheep and cattle, individual QTL locations and bovine QTL were extracted from the literature, and were loaded into the ovine genome database, which can be browsed at http://crcidp.vetsci.usyd.edu.au/cgi-bin/gbrowse/oaries_genome/. While a meta-assembly of all known QTL was performed as outlined in previous papers [18,38], no meta-score was calculated for lactation persistency due to the inconsistency of trait definitions of persistency and the inconsistency of QTL positions. However, as publications on persistency become available in the future, we plan to update the ovine genome database to facilitate a full meta-assembly.

## Results

### Analysis of lactation persistency

The Wood model parameters were standardised against the predicted lactation curves for the following fixed effect classifications: five years of age, singleton birth type, third parity, twice milking/day and fourth season for milk yield; five years of age, second parity, and twice milking/day for protein yield; three years of age, second parity, twice milking/day and singleton birth type for fat yield; three years of age, twice milking/day, singleton birth type and second parity for useful yield; and seven years of age for lactose and somatic cell score yield. The factors used for standardisation differed across the lactation traits, as not every factor had a significant effect on all traits. Lactation persistency was highest for useful yield (0.65), while daily milk yield persistency had the lowest average value (0.23) (Table 1). Similarly, extended lactation had the highest average for useful yield, followed by fat and protein, and was lowest for milk yield (Table 1). Most of the lactation persistency characteristics for different milk traits showed low to moderate phenotypic correlations, with the exception of persistency of protein yield, which was highly correlated to persistency of fat and somatic cells (Table 2). The highest correlations between lactation persistency and extended lactation were observed for milk, lactose and somatic cell yields (0.79-0.89, *P *≤ 0.01) (Table 2).

**Table 1:** Descriptive statistics of the persistency and extended lactation traits used in this study

Trait	Milk composition	*n*	mean	SD	min	max
	Milk	174	0.23	0.13	0.03	0.72
	Protein	162	0.47	0.15	0.14	0.99
Persistency	Fat	160	0.40	0.14	0.10	0.79
	Lactose	157	0.35	0.18	0.00	0.71
	Somatic cells	159	0.43	0.07	0.23	0.63
	Useful yield	162	0.65	0.12	0.28	0.91

	Milk	174	0.37	0.15	0.10	0.81
	Protein	161	0.89	0.23	0.42	1.97
Extended lactation	Fat	155	0.91	0.18	0.47	1.45
	Lactose	154	0.54	0.33	0.00	1.34
	Somatic cells	159	0.56	0.07	0.38	0.75
	Useful yield	144	1.09	0.30	0.48	1.75

Shown are the number of sheep with observations (*n*), the average (mean), standard deviation (SD), lowest (min) and highest (max) values of the variables calculated by fitting the Wood model using the nonlinear mixed model procedure in 'R'.

**Table 2:** Phenotypic correlations among lactation persistency and extended lactation traits

Trait	Extended lactation						Lactation persistency				
	
	MY	PY	FY	UY	LY	SCY	MY	PY	FY	UY	LY
Extended lactation protein	-0.18										
Extended lactation fat	-0.25	0.67									
Extended lactation useful yield	-0.12	0.72	0.69								
Extended lactation lactose	-0.06	0.01	0.13	0.08							
Extended lactation somatic cell	-0.04	0.62	0.64	0.83	0.08						
Milk persistency	0.88	-0.20	-0.28	-0.12	-0.07	-0.02					
Protein persistency	0.18	0.54	0.45	0.54	0.27	0.59	0.23				
Fat persistency	0.21	0.18	0.28	0.31	0.21	0.31	0.25	0.64			
Useful yield persistency	0.02	0.29	0.42	0.45	0.44	0.39	0.01	0.55	0.51		
Lactose persistency	0.04	0.01	0.11	0.10	0.85	0.09	0.01	0.39	0.36	0.54	
Somatic cell persistency	0.15	0.44	0.46	0.62	0.16	0.78	0.23	0.77	0.59	0.49	0.30

Correlations are shown for lactation persistency and extended lactation of milk (MY), protein (PY), fat (FY), lactose (LY), useful yield (UY) and somatic cell score (SCY) yields. Phenotypic correlations greater than 0.22 are highly significant (*P *≤ 0.01), greater than 0.17 are significant (*P *≤ 0.05), and smaller than 0.15 are not significant (*P *≥ 0.1).

### Putative QTL for lactation persistency

**Figure 1:**
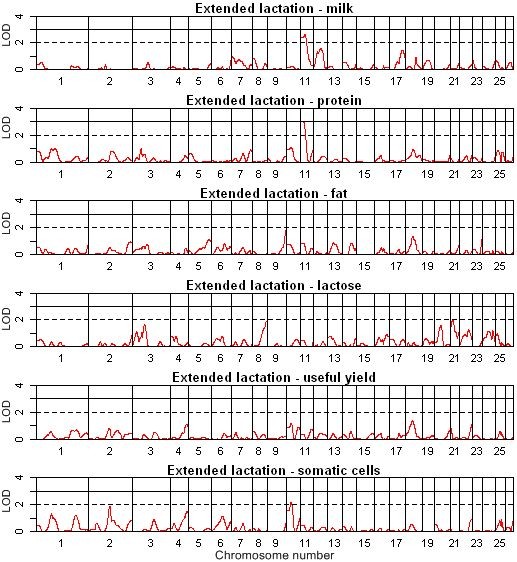
**QTL map of the entire genome for extended lactation of milk and milk composition**.

**Figure 2:**
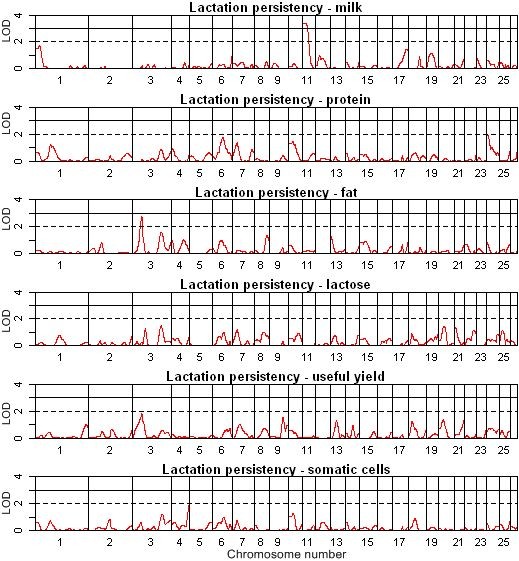
**QTL map of the entire genome for milk and milk composition persistency**.

Four significant (LOD > 2.0) and one highly significant (LOD > 3.0) QTL were detected on chromosomes 3, 10 and 11 using single QTL analysis across both methods. Six suggestive (1.7 ≤ LOD < 2.0), four significant (2.0 ≤ LOD < 3.0) and one highly significant (LOD ≥ 3.0) QTL were identified using QTL-MLE (Table 3, Figure 1, 2). Using QTL Express, eight suggestive (chromosome-wide *P *< 0.05), six significant (chromosome-wide *P *< 0.01) and two highly significant (experiment-wide *P *< 0.05) QTL were identified [see Additional file 2]. On OAR11, QTL for lactation persistency, extended lactation of milk, and extended lactation of protein were detected using both linkage methods. QTL for lactation persistency and extended lactation of milk and lactose were also identified on chromosome 17 and 21, respectively, but these were not verified by using QTL-MLE. We hypothesised that persistency of different lactation traits and extended lactation are likely to be controlled by different genes, with the possible exception of a common gene located on OAR11, which controls extended lactation for milk and protein yield, as well as milk persistency (Table 3).

**Table 3:** Results of the QTL analysis using QTL-MLE

OAR	Trait	Position		LOD	QTL effect	
		QTL [CI] in cM	Marker		Est	SD
2	Extended lactation somatic cells	175 [157-184]	TGLA10-BM81124	1.9*	-0.04	-0.55
3	Fat persistency	76 [62-83]	BM8118-BMS710	2.8**	-0.09	-0.60
4	Somatic cells persistency	146.6 [131-147]	OARHH35-MCM73	2*	-0.04	-0.49
8	Extended lactation lactose	116.4 [77-123]	BM3215-BMS1967	1.9*	-0.18	-0.55
9	Extended lactation fat	154 [136-154]	BM4513-RJH1	1.9*	0.09	0.48
10	Extended lactation somatic cells	39.9 [28-55]	MNS64-OARHH41	2.2**	0.04	0.58
11	Extended lactation milk	39.3 [29-58]	HEL10-BM17132	2.6**	-0.09	-0.64
11	Milk persistency	32.3 [29-50]	HEL10-BM17132	3.4***	-0.09	-0.64
11	Extended lactation protein	29.3 [29-45]	HEL10-BM17133	3**	0.14	0.59
21	Extended lactation lactose	19 [0-49]	BMC2228-CSSM013	2*	0.23	0.69
24	Protein persistency	6.1 [5-30]	OARJMP29-BMS744	2*	0.07	0.49

Information of the average QTL position (QTL) and 1-LOD drop-off confidence interval (CI) in cM; flanking markers of the peak; LOD score with respective significance level [*suggestive QTL (1.7 ≤ LOD < 2.0), **significant QTL (2.0 ≤ LOD < 3.0), ***highly significant QTL (LOD ≥ 3.0)]; estimated QTL effect (Est) and standardised QTL effect (SD) [estimated effect difference (Awassi-Merino) relative to the estimated phenotypic standard deviation] are presented.

Most of the QTL identified here showed positive (not necessarily favourable) effects for the Awassi allele, compared with the Merino allele. The largest effect was identified for extended lactation of lactose (0.69 SD) on OAR21. Persistency and extended lactation of milk showed a negative effect of the Awassi allele (-0.64 SD) on OAR11 (Table 3). The average QTL heritability, expressed as the proportion of the phenotypic variance accounted for by the QTL, and standardised QTL effects were slightly higher for extended lactation (5.48% and 0.58 SD) than for lactation persistency (5.11% and 0.56 SD) (Table 3) and [see Additional file 2]. The QTL heritability reached its highest value for fat traits and its lowest for useful yield (the QTL heritability is shown in Additional file 2). The standardised QTL effects were similar for all traits and reached values between 0.48 and 0.69 phenotypic SD. (Table 3). The greatest standardized QTL effects were found for somatic cells, and the smallest for lactose [see Additional file 2].

Using the two-QTL model for all traits across all chromosomes, evidence for two pairs of QTL was found on OAR10 (Table 4). For extended lactation of useful yield, neither of the two QTL were detected using single-QTL methods, while for extended lactation of somatic cells, one of the QTL positions was identified in both single-QTL approaches. Both pairs of QTL were in repulsion phase of opposite (unequal) effect and were located in separate flanking marker intervals, giving support for these being separate QTL but this needs to be validated in a separate study.

**Table 4:** Results of the two-QTL analysis using QTL Express

OAR	Trait	Position (cM)		Flanking marker		*F*-value		SD (SE)	
		QTL A	QTL B	QTL A	QTL B	2vs0	2vs1	QTL A	QTL B
10	Extended lactation somatic cells	12	84	MNS64-OARHH41	TGLA441-OARDB3	8.36*	6.49*	0.61 (0.18)	-0.52 (0.21)
10	Extended lactation useful yield	13	62	MNS64-OARHH41	ILSTS056-TGLA441	6.06*	6.92*	0.56 (0.20)	-0.52 (0.20)

Shown are the peak position of QTL A and QTL B; flanking markers of both peaks; *F*-value and significant threshold (*chromosome-wide *P *< 0.05) reached under 2 vs 0 QTL (*F*-statistic for testing two QTL vs no QTL on chromosome), or 2 vs 1 QTL (*F*-statistic for testing two QTL vs one QTL on chromosome); standardised QTL effect (SD) and standard error (SE).

### Meta-assembly

Two genome-wide and two partial bovine QTL studies [10,12,28,29] were summarised in the meta-assembly; results from two genome-wide association studies were not loaded into the ovine genome database [39,40]. Until now, no QTL study for lactation persistency in sheep has been reported. QTL for milk and milk composition persistency in cattle have been reported on chromosomes 1, 2, 6, 9, 14, 15, 17, 18, 21, 22, 25 and × using a granddaughter design of German Holstein dairy cattle [28]. QTL were also identified for shape of the lactation curve for milk, protein, fat and SCS in U.S. Holstein cattle, confirming the QTL on chromosomes 6, 14, 21 and 22 identified in German Holstein Friesians, and finding additional QTL regions on chromosomes 3 and 7 [29], while Weller et al. [10] could not verify a QTL for milk persistency on BTA7. Other QTL have been reported on BTA11 and 17 [12]. All QTL, except those on BTA15, 22 and 25, were confirmed by association studies, and additional associations have been identified on all other bovine chromosomes [39-41].

Four bovine QTL for lactation persistency traits have been reported on chromosomes that are orthologous to OAR3, 9, 17 and 24, where we identified QTL for these traits in sheep. The QTL on OAR9 and OAR17 aligned to QTL for the same milk persistence characteristics reported on the orthologous bovine chromosomes (BTA14 and BTA17, respectively) [12,29]. The QTL for milk persistency on BTA14 had its peak within the region of the *DGAT1 *gene. The QTL identified for fat yield extended lactation on OAR9 (BM4513-RJH1) in our study aligned to the bovine QTL for fat persistency at 139 cM (BM4513-BL1036).

## Discussion

This paper is a continuation of our previous study of lactation characteristics in a sheep population [18]. It extends the application of the Wood model using a nonlinear mixed model to describe lactation curves by deriving persistency and extended lactation measures for milk, protein, fat, lactose, useful yield and somatic cell yield. It identifies novel QTL for both persistency and extended lactation traits and compares linkage and genome-wide association studies for these traits across sheep and cattle.

### Lactation persistency and extended lactation

Measures of persistency and extended lactation can be used to define the shape of lactation curves, the former focusing on shape within the conventional lactation length (say 100 days in sheep, 305 days in cattle) and the latter on sustained production after this period. Both these measures are useful indicators for practical management applications. Many other measures of lactation curve shapes have been described in the literature, some based on parameters in complex lactation curve models [11], others related to the time to reach a certain percentage of total milk production [2] or using smoothing cubic splines [39]. It has been suggested that persistency measures that are uncorrelated with total milk yield, for example using the shape of lactation curve independent of level of production, allows simultaneous selection for total lactation yield and persistency [4,42].

To our knowledge, there are no comparable studies of lactation persistency of different milk components in sheep, but in a study in cattle, the highest persistency was found for protein yield, followed by fat, and the lowest for milk yield [4]. We could confirm the lower values for persistency of milk yield in sheep, but the persistency of fat yield was higher than the persistency of protein yield. Considering that the major use of sheep milk is for producing cheese, protein and fat yields, and their combination in the form of useful yield, are important traits, and their persistency over the length of the lactation, enables a more sustained outflow of milk for cheese production [43].

A study in cattle [44] reported negative phenotypic correlations of total merit traits (namely lifetime net merit, cheese merit and fluid merit) with persistency of SCS. In the same study, it was also found that phenotypic correlations of milk, fat, and protein with SCS were negative, and that persistency of SCS had a close to zero correlation with SCS. They also found that persistency of SCS had low phenotypic correlations with milk, fat, and protein yield across breeds, but these findings were not observed for most of the traits in our study. It is difficult to resolve if this represents a difference between cattle and sheep, or if it is due to differences in the definition of lactation persistency [44].

While there is a reasonable amount of literature describing lactation persistency, particularly in cattle, little has been published in relation to extended lactation. The general assumption is that persistency and extended lactation represent similar traits. However, some studies have specifically aimed at describing extended lactation, e.g. Grossman and Koops [45], who investigated appropriate modelling techniques, and Haile-Mariam and Goddard [14], who reported on genetic parameters associated with extended lactation in cattle. While conventional lactation curve modelling studies may be extrapolated to predict extended lactation characteristics, empirical data on actual yields is needed to allow model fits to be assessed. The current study is based on observed milk yield beyond the standard lactation length in sheep (day 100), as are the two cattle studies reported above (beyond day 305).

### Putative QTL

As shown in the first three papers of this series [17,29,37], the results from the maximum likelihood approaches to QTL detection (QTL-MLE) were in good agreement with those of the least-squares methodology of QTL Express [37]. However, it should be pointed out that QTL-MLE is based on fitting a finite mixture model, unlike regression-based methods, which mirrors the underlying Mendelian segregation process of the putative QTL [46].

To our knowledge, this is the first study describing a whole genome-scan for detecting QTL for lactation persistency and extended lactation of milk and milk composition in sheep. The results suggest the existence of important genomic regions for these traits but overall, the different traits are influenced by many different QTL (Tables 3, additional file 2). In a previous meta-assembly of published QTL reports, QTL for milk yield were identified on ovine chromosomes 1 to 3, 6, 9, 14, 16, 20, 22, and 24 [18]. However none of the regions identified for lactation persistency and extended lactation of milk on OAR11, 12, or 17 corresponded with these findings and only the QTL for milk persistency on OAR-17 was located in the comparative region to BTA17, where a QTL for milk persistency was identified in dairy cattle [12].

Among the two QTL identified for protein persistency and extended lactation on OAR11 and 24, only the QTL on OAR24 aligned to a QTL for milk persistency on the comparative bovine chromosomes 25 [28]. The QTL for lactation persistency and extended lactation of fat on OAR3 aligned with the QTL regions for average fat yield and energy persistency on BTA3 [28]. The QTL on OAR9 aligned with QTL for fat persistency on BTA14 at 139 cM. Rodriguez-Zas et al. [28] also reported on a QTL for the shape of the bovine lactation curve of fat at the proximal end of BTA14, which overlaps with the position of the *DGAT1 *gene [28,29]. The previous meta-assembly [18] also showed strong evidence of QTL for fat yield on both ovine chromosomes 3 and 9. Given the lack of QTL studies in both sheep and cattle for lactation persistency and extended lactation, the power of comparative mapping cannot be evaluated.

Among the three QTL regions for persistency and extended lactation of somatic cells identified on OAR2, 4 and 10, only the QTL on chromosome 2 mapped to a previously reported QTL in an ovine meta-assembly [18]. The QTL on OAR2 and 4 were supported by an association study for milk persistency [39]. Our QTL findings for useful yield and lactose yield are novel since there is no other published study.

From this study there does not appear to be a single major gene that regulates lactation persistency and extended lactation. Despite the reduced power to detect QTL for lactation traits since they are expressed in females only, the power of this experiment was sufficient to detect QTL with a minimum effect size of 0.4 SD. Such QTL may have utility in marker-assisted selection in the Awassi-Merino population used here. The Awassi breed has been used extensively to improve milk production through upgrading or crossbreeding programs and therefore QTL detected in the Awassi breed may also support marker-assisted introgression programs. Before such programs can be adopted, the QTL need validation in terms of effect sizes and location. The QTL findings also provide a foundation for targeted fine-mapping using SNP before positional candidate genes can be investigated. In particular, the QTL on OAR11 with effects on milk persistency and on extended lactation for milk and protein yield warrants further investigation.

## Conclusion

This paper presents measures of lactation persistency and extended lactation in dairy ewes using the Wood lactation curve model. For the first time, QTL were identified for lactation persistency and extended lactation for milk components in a sheep resource population. It is shown that the QTL for lactation persistency and extended lactation for milk components (protein, fat, lactose, useful yield, SCS) differ from QTL for the corresponding yield traits. Furthermore, for many traits, the QTL affecting persistency are different from those affecting extended lactation. While a number of QTL of moderate effect size have been mapped, no individual QTL of major effect has been identified for persistency or extended lactation. Some QTL affecting similar traits in cattle and sheep were found to map to the same homologous regions. However, our ability to undertake this species comparison is limited by the relatively large size of support intervals in the sheep QTL study and by different trait definitions across the studies.

## Competing interests

The authors declare that they have no competing interests.

## Authors' contributions

EJ ran the final data and QTL analyses, and took the lead role in the preparation of the manuscript. PCT developed the statistical methodology for the lactation curve analysis and QTL methodology, implemented the QTL-MLE program, and contributed to the manuscript's preparation and the overall design. EJSH and DMcG carried out the analysis of the lactation curves, developed the models and helped drafting the manuscript. MKL ran the early stage QTL analyses, was responsible for the data assembly, and the phenotypic analysis. HWR had principal lead in the overall design, undertook the project management, and was involved in analysing the data and writing the manuscript. All authors read and approved the final manuscript.

## Supplementary Material

Additional file 1 **Number of observations at different stages of the lactation**. Number of observations during the lactation: the number of observations dropped from *n *= 565 at day 100 to *n *= 334 at day 150 and *n *= 172 at day 200 of lactation. Click here for file

Additional file 2 **Results of the QTL analysis using QTL Express**. Information on the average QTL position (Peak) and the confidence interval (CI) in cM; *F*-value and respective significance threshold [*chromosome-wide significance level *P *< 0.05, **chromosome-wide significance level *P *< 0.01, ***experiment-wide significance level *P *< 0.05, ****experiment-wide significance level *P *< 0.01]; standardised QTL effect (SD) with standard error (SE); and phenotypic variance explained by the QTL are presented. Click here for file
