# Supplementary material for: Mapping quantitative trait loci (QTL) in sheep. IV. Analysis of lactation persistency and extended lactation traits in sheep
Source: Genet Sel Evol. 2011 Jun 21;43(1):22. doi: 10.1186/1297-9686-43-22 (PMC3152874; doi:10.1186/1297-9686-43-22)

Additional file 1

File format: PDF

Title: Number of observations at different stages of the lactation

Description: Number of observations during the lactation: the number of observations dropped from n = 565 at day 100 to n = 334 at day 150 and n = 172 at day 200 of lactation.

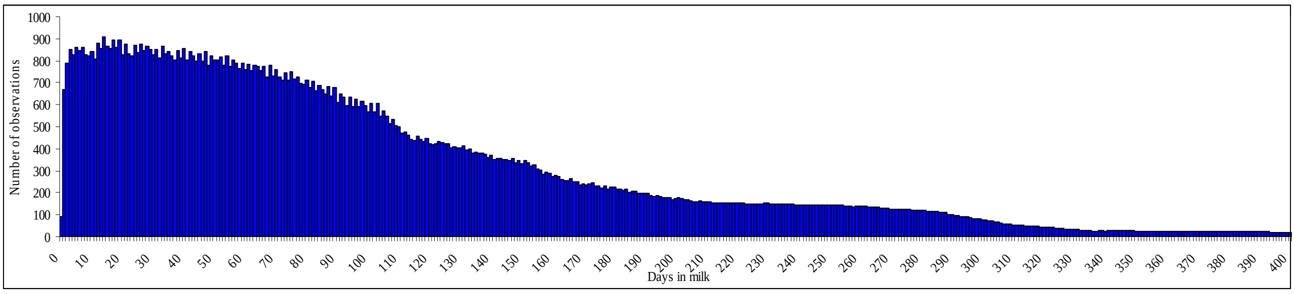

Supplement: Additional file 1 — Number of observations at different stages of the lactation. Number of observations during the lactation: the number of observations dropped from n = 565 at day 100 to n = 334 at day 150 and n = 172 at day 200 of lactation. [file 1297-9686-43-22-S1.PDF]
