# Supplementary material for: Mapping quantitative trait loci (QTL) in sheep. IV. Analysis of lactation persistency and extended lactation traits in sheep
Source: Genet Sel Evol. 2011 Jun 21;43(1):22. doi: 10.1186/1297-9686-43-22 (PMC3152874; doi:10.1186/1297-9686-43-22)
Supplement: Additional file 2 — Results of the QTL analysis using QTL Express. Information on the average QTL position (Peak) and the confidence interval (CI) in cM; F-value and respective significance threshold [*chromosome-wide significance level P < 0.05, **chromosome-wide significance level P < 0.01, ***experiment-wide significance level P < 0.05, ****experiment-wide significance level P < 0.01]; standardised QTL effect (SD) with standard error (SE); and phenotypic variance explained by the QTL are presented. [file 1297-9686-43-22-S2.PDF]

Additional file 2

File format: PDF

Title: Results of the QTL analysis using QTL Express

Description: Information on the average QTL position (Peak) and the confidence interval (CI) in cM; *F*-value and respective significance threshold [*chromosome-wide significance level *P* < 0.05, **chromosome-wide significance level *P* < 0.01, ***experiment-wide significance level *P* < 0.05, ****experiment-wide significance level *P* < 0.01]; standardised QTL effect (SD) with standard error (SE); and phenotypic variance explained by the QTL are presented.

OAR	Trait	Peak [CI] in cM	Flanking markers	*F*-value	SD (SE)	var
3	Fat persistency	75 [64-314]	BM8118-BMS710	13.3**	-0.64 (0.14)	7.3
4	Somatic cells persistency	135 [2-135]	OARHH3-MSM73	9.4*	-0.57 (0.14)	5.1
8	Extended lactation lactose	102 [22-106]	BM3215-BMS1967	9.7**	-0.57 (0.19)	5.5
9	Extended lactation fat	146 [22-146]	BM4513-RJH1	8.7*	0.51 (0.17)	4.9
10	Protein persistency	8 [0-68]	MNS64-OARHH4	6.9*	0.47 (0.18)	3.6
10	Extended lactation somatic cells	12 [0-84]	MNS64-OARHH4	9.9**	0.55 (0.14)	5.4
11	Milk persistency	0 [0-19]	HEL10-BM17132	17.6****	-0.62 (0.16)	8.9
11	Extended lactation milk	10 [0-28]	HEL10-BM17132	14.1***	-0.70 (0.21)	7.1
11	Extended lactation protein	40 [0-76]	HEL10-BM17132	13.3**	0.60 (0.16)	7.3
12	Extended lactation milk	53 [19-106]	TGLA53-MCMA52	8.0*	0.55 (0.21)	4.0
13	Useful yield persistency	42 [0-97]	MCM152-HUJ616	6.7*	0.51 (0.17)	3.5
17	Extended lactation milk	85 [32-96]	BM7136-TGLA322	10.8**	0.76 (0.21)	5.5
17	Milk persistency	89 [32-96]	BM7136-TGLA322	11.3**	0.77 (0.23)	5.7
21	Lactose persistency	5 [0-78]	BMC2228-CSSM013	5.9*	0.51 (0.23)	3.1
21	Extended lactation lactose	17 [0-78]	BMC2228-CSSM013	7.4*	0.63 (0.22)	4.1
24	Protein persistency	4 [0-96]	OARJMP29-BMS744	7.2*	0.48 (0.18)	3.7
